# The feasibility and safety of cocktail treatment of triple anti-inflammatory agents loaded with gelatin sponge promotes early recovery after posterior percutaneous endoscopic cervical discectomy

**DOI:** 10.1186/s13018-022-03178-2

**Published:** 2022-05-26

**Authors:** Peng Zou, Xiaoping Zhang, Rui Zhang, Jun-Song Yang, Lei Chu, Xiang-Fu Wang, Jian-Min Wei, Xin Chai, Yuan-Ting Zhao, Bo Liao

**Affiliations:** 1 Department of Orthopedics, Tangdu Hospital, Air Force Medical University, Xi’an, Shaanxi China; 2 Department of Spine Surgery, Honghui Hospital, Xi’an Jiaotong University, Xi’an, Shaanxi China; 3 Department of Minimally Invasive Spine Surgery, The Second Affiliated Hospital of Chongqing Medical University, No. 76 Linjiang Road, Chongqing, 400010 China; 4 Department of Spinal Minimally Invasive Surgery, Gansu Provincial Hospital of Traditional Chinese Medicine, Lanzhou, Gansu China; 5 Department of Spine Surgery, Baoji City Hospital of Traditional Chinese Medicine, Shaanxi, China

**Keywords:** Posterior percutaneous endoscopic cervical discectomy, Cocktail treatment, Nerve root block, Gelatin sponge

## Abstract

**Purpose:**

To investigate whether a cocktail therapy of dexamethasone, ropivacaine, dexmedetomidine, and vitamin B12 can achieve satisfactory pain relief and promote early functional recovery after PPECD.

**Methods:**

Eighty single-level patients with CDH who received PPECD were retrospectively divided into two groups: the cocktail and control groups. Clinical data were recorded and evaluated by a dedicated physician who was not involved in the patient’s treatment. The primary clinical outcomes included visual analog scores (VASs) for upper limber pain and neck disability index (NDI) scores. The follow-up time points were preoperatively and postoperative 1 week, 1 month, 3 months, 6 months, and 12 months. The modified MacNab criteria was used to evaluate the surgical effect of the last follow-up.

**Results:**

The follow-up data of 74 cases were complete, except 6 cases lost to follow-up. There was no significant difference between the two groups in demographics, duration of symptoms, operation stage (*p* > 0.05), and operation time (80.5 ± 5.5 vs. 81.5 ± 3.5 min). The VAS in the upper limbs pain was significantly higher postoperatively than preoperatively in both groups (*p* < 0.05). The cocktail group had a lower VAS than the control group 1 week postoperatively (*p* < 0.05); however, VAS not different between groups at the remaining time points. The NDI scores were significantly better postoperatively than preoperatively, and no significant differences were seen when comparing nodes at postoperative follow-up (*p* > 0.05). In the control group, two cases with foraminal stenosis were found to have unrelieved pain in the early postoperative period, but the pain was relieved at the final follow-up and did not convert to open decompression surgery.

**Conclusions:**

Cocktail treatment, in which a drug sustained-release material made of gelatin sponge was impregnated with dexamethasone, ropivacaine, dexmedetomidine and vitamin B12, facilitates pain relief and early postoperative recovery after PPECD.

## Background

In the treatment of non-median cervical disc herniation (CDH), compared with traditional classic surgery, anterior cervical decompression and fusion (ACDF) [1] posterior percutaneous endoscopic cervical discectomy (PPECD) achieves similar surgical effects, but induce less damage, have a shorter recovery period and good postoperative biomechanical maintenance, and eliminates the need for internal fixation [2–5]. Among PPECD related complications, including postoperative epidural hematoma, dura tear, total spinal cord anesthesia, and spinal cord injury caused by continuous pressure washing or intraoperative manipulation [4, 6], nerve root dysfunction, such as upper limb radiation pain, dysesthesia, or weakness, is the most common. As a result of alleviating such complications, in addition to modifying surgical techniques, administering anti-inflammatory drugs is the popularized method of relieving nerve root dysfunction symptoms. Different from oral, intravenous, and intramuscular administration, intraoperative local injection around the nerve via a working channel is more targeted. In addition to improving local drug concentration, side effects related to oral and intravenous medication are also reduced, which is more in line with the concept of safe and efficient surgery under minimally invasive spine surgery. We first [7] introduced the "cocktail treatment," in which a gelatin sponge was impregnated with ropivacaine, dexamethasone, and vitamin B12 into percutaneous endoscopic lumbar disectomy (PELD), and observed that the method indeed shortens the duration of recovery and lends better clinical outcomes. Unlike PELD, PPECD has limited manipulation space, and once anesthesia-related complications, such as total spinal cord anesthesia [7], occur, the results will be disastrous. Therefore, it is necessary to evaluate the feasibility and safety of the cocktail treatment of triple anti-inflammatory agents. We hypothesized that a cocktail therapy of dexamethasone, ropivacaine, dexmedetomidine, and vitamin B12 can achieve satisfactory pain relief and promote early functional recovery after PPECD; accordingly, we conducted this study to validate this hypothesis. Moreover, we used artificial biological materials composed of gelatin sponge to make drug slow-release material (DSM) to prolong the action time of drugs.

## Methods

### Patients

This study was approved by the institutional review board of all included hospitals, and informed consent from each patient was obtained. From January 2018 to June 2020, 80 single-level patients with CDH who received PPECD in our hospital were retrospectively included. Patients were equally divided into two groups: the cocktail and control groups.

All operations were performed by the same physician, who has experience in more than 500 spinal endoscopic surgeries. The demographic characteristics and surgical levels of patients are shown in Table 1.

**Table 1:** Patient demographics and surgical characteristics of both groups

	Cocktail group	Control group	P value
Total (n)	38	36	
Age (years)	44.7 ± 6.8	45.4 ± 7.5	0.689
Gender (male:female)	20:18	18:18	0.821
Body Mass Index (%)	23.5 ± 1.7	24.2 ± 2.1	0.135
Operation time (min)	80.5 ± 5.5	81.5 ± 3.5	0.413
Surgical levels			0.886
C3–C4	2	3	
C4–C5	7	8	
C5–C6	16	14	
C6–C7	13	11	

Inclusion criteria were (1) single-level foraminal or lateral cervical disc herniation compromising the nerve root with neurological deficit; (2) unilateral cervical radiculopathy with arm pain with or without loss of sensory or motor function; and (3) failure of or worsening symptoms for at least 6 weeks of conservative therapy. Exclusion criteria were (1) obvious instability or malformation, suspected infection, or tumor in the cervical spine; (2) multiple-level cervical disc herniation with severe degeneration; (3) medial type disc herniation; and (4) allergy to vitamin B12, ropivacaine dexmedetomidine, and dexamethasone.

### Surgical technique and cocktail treatment

**Fig. 1:**
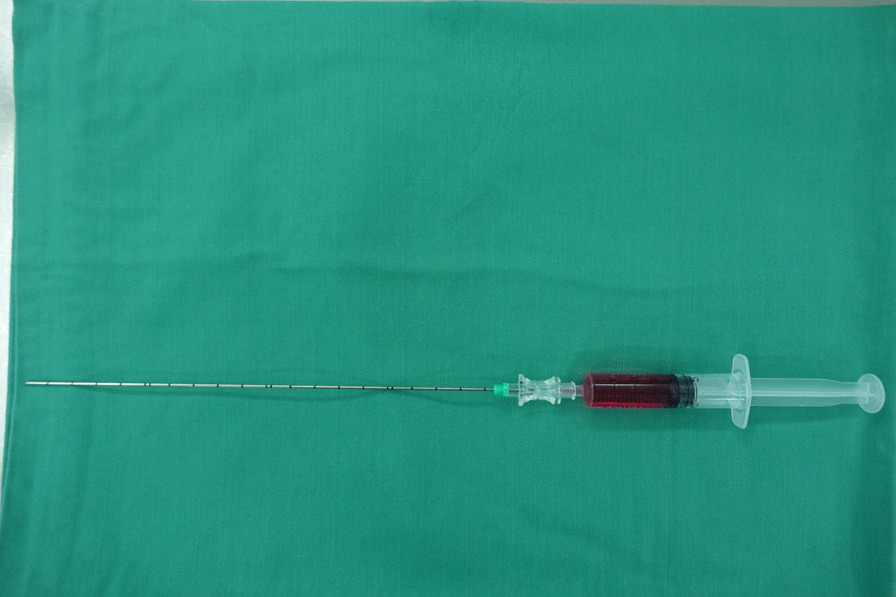
The mixture of 4 drugs containing dexamethasone, ropivacaine, dexmedetomidine and vitamin B12

**Fig. 2:**
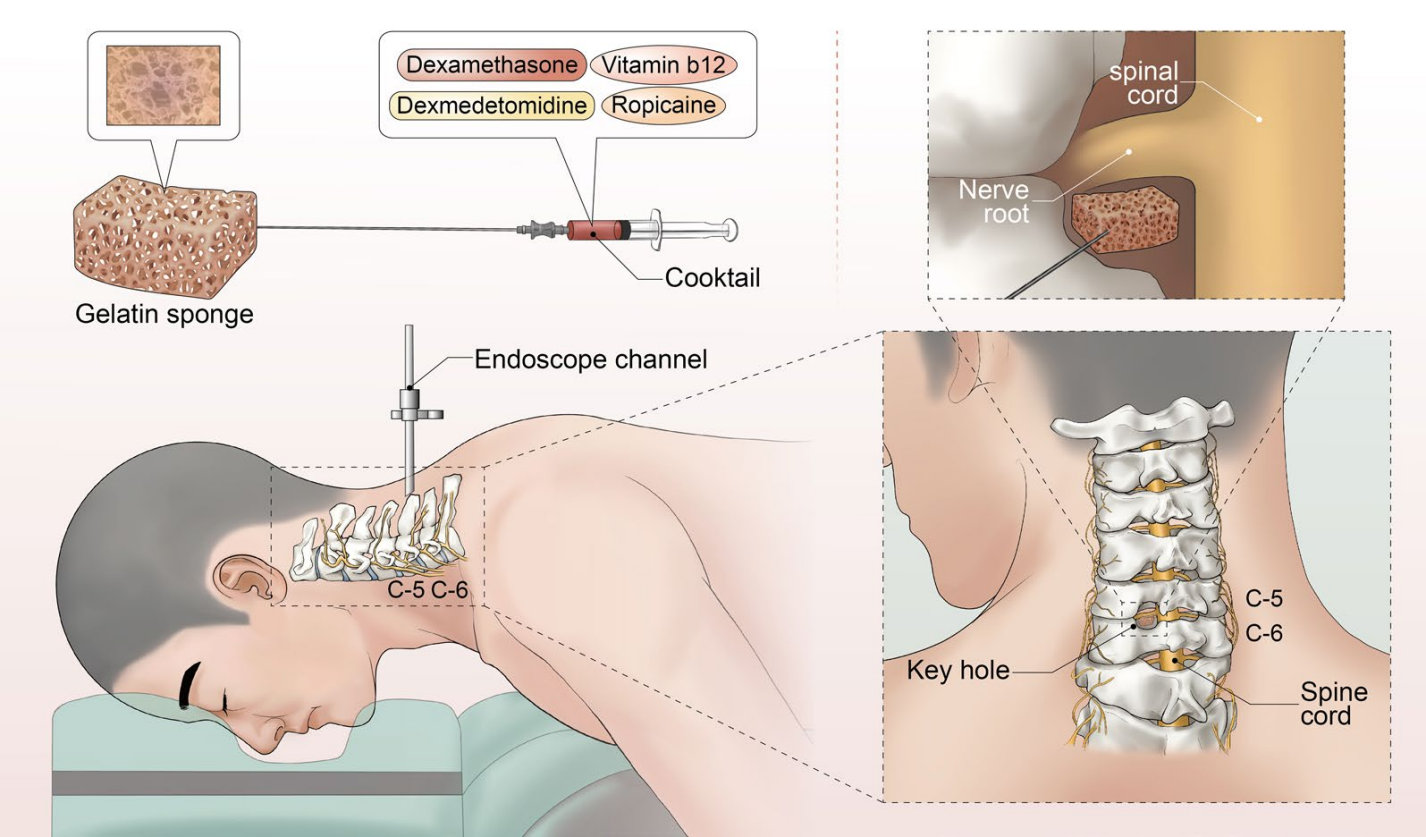
Schematic diagram of cocktail therapy. When decompression was fully determined, a suitable size gelatine sponge was place on the dorsal side of the nerve root through the working cannula. A syringe with a long needle was used to inject the 3 mL of mixed liquid into the gelatin sponge

The detailed procedure is in accordance with that reported in previous studies [4, 6, 8]. After general anesthesia, the patient was placed in the prone position, and the neck was slightly flexed. The preparation of the cocktail is all done by the anesthetist. The cocktail is formulated as follows: dexamethasone injection 15 mg (3 ml); 1% ropivacaine injection 8 mg (0.8 ml); dexmedetomidine 0.2 µg/Kg; and vitamin B12 0.5 mg (2 ml), diluted to 8 ml (Fig. 1). The target level of the vertebra was demarcated by fluoroscopy, an incision of about 8–9 mm was made in the affected side. Following needle placement, sequential dilation, and working sheath establishment, the endoscope was inserted through the working sheath (Joimax®, Germany). The bevel of the work sheath was directed towards the medial side. Radiofrequency and grasp were used to clean the soft tissues of the facet joint and lamina to expose the "V" point structure. Thereafter, the bone was removed at the the "V" point using an endoscopic burr or osteotome to expose the deep ligamentum flavum. After the ligamentum flavum and mesangium were cut with a basket forceps, the deep frontal dural sac and nerve roots were exposed. The nerve was pulled to confirm the protrusion of the nucleus pulposus. After the nucleus pulposus tissue was removed, the nerve root was pulled to detect whether there were osteophytes located at the ventral uncinate joint of the nerve root. Bony decompression was further achieved with a grinding burr or osteotome. Decompression was fully determined by the free movement of nerve roots from the proximal to distal outlet zones. In the cocktail group, before the end of operation, we first cut the DSM to a suitable size (approximately 1.0 cm × 0.5 cm) and subsequently used endoscopic forceps to place it on the dorsal side of the nerve root. A syringe with a long needle was used to inject the 3 mL of mixed liquid into the gelatin sponge (Fig. 2). The control group did not undergo the cocktail injection step. All patients were advised to wear a neck collar for at least 4 weeks.

Clinical data were recorded and evaluated by a dedicated physician who was not involved in the patient’s treatment. The primary clinical outcomes included visual analog scores (VASs) for upper limber pain and neck disability index (NDI) scores. (NDI (%) = [total item score/(number of items × 5)] × 100%). The follow-up time points were preoperatively and postoperative 1 week, 1 month, 3 months, 6 months, and 12 months. The main clinical complications were TSA, dural tear, motor dysfunction, and wound infection. The modified MacNab criteria was used to evaluate the surgical effect of the last follow-up.

### Statistical analysis

R version 3.5.3 was used for statistical analysis. An analysis of variance was used to compare the means of continuous variables with normal distributions. In cases of a normal distribution, continuous variables were summarized by mean ± standard deviation. Student t-tests were used for comparisons between independent samples; paired t-tests were used for comparisons between matched samples. If not normally distributed, the median (interquartile range) was used, and the Mann–Whitney U test was applied. Categorical data were expressed as numbers and percentages and assessed by the chi-square test.

## Results

**Fig. 3:**
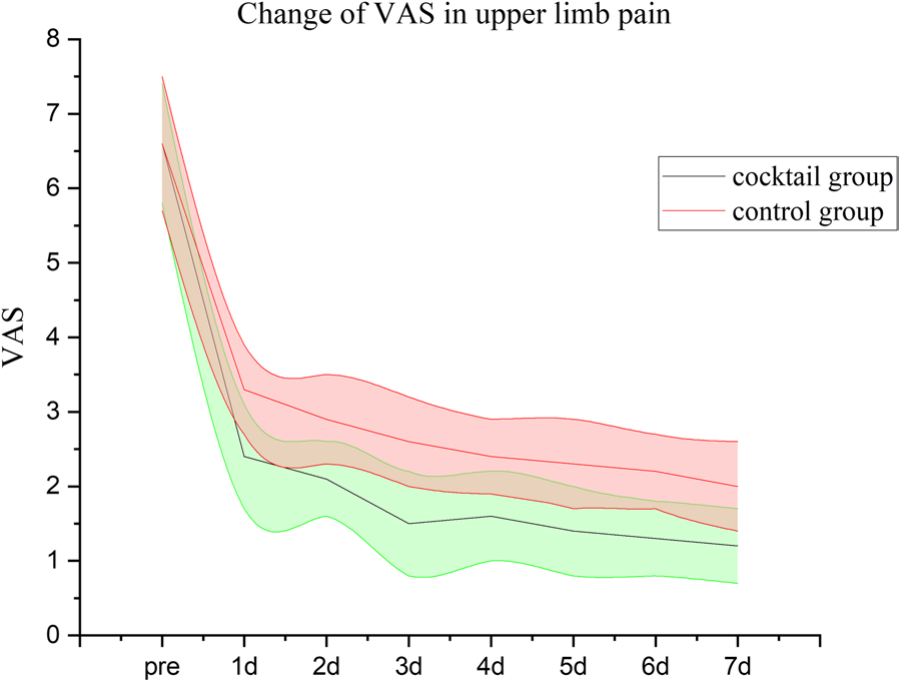
Short-term visual analog scale (VAS) score of upper limb pain from preoperatively to postoperative 7 days

**Fig. 4:**
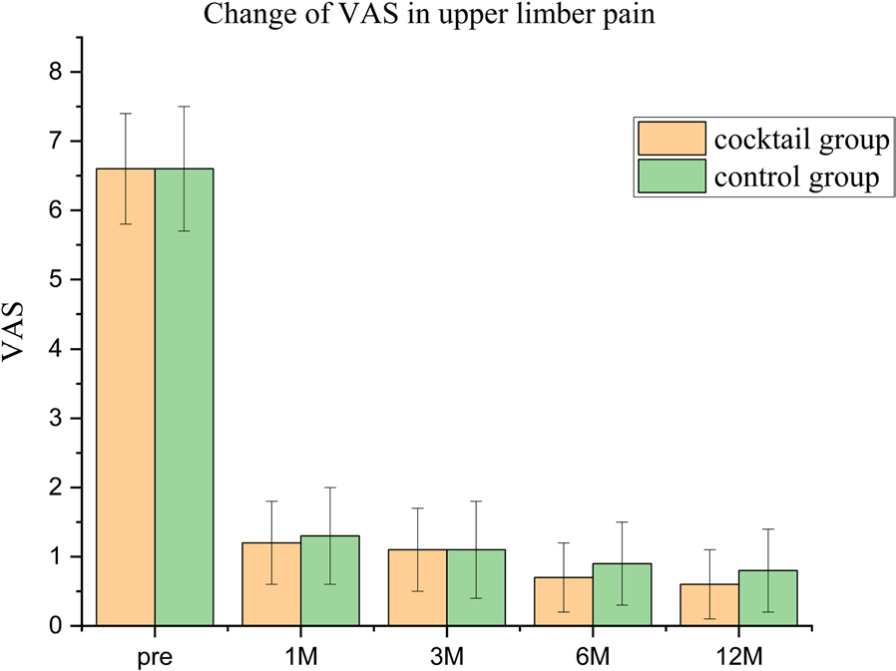
Long-term visual analog scale (VAS) score of upper limb pain from preoperatively to postoperative 12 months

**Fig. 5:**
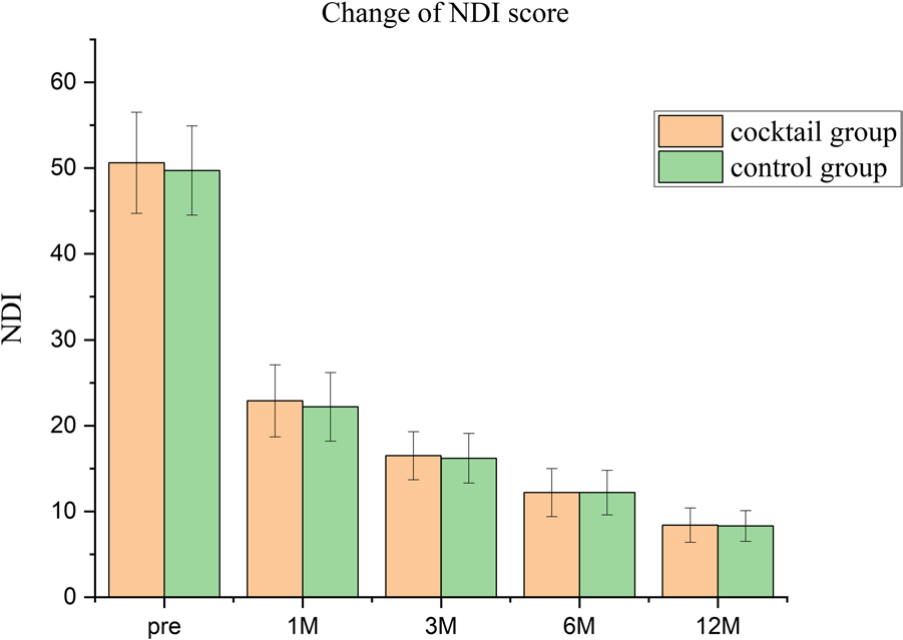
Long-term neck disability index (NDI) from preoperatively to postoperative 12 months

The follow-up data of 74 cases were complete, except 6 cases lost to follow-up. There were 38 patients in the cocktail group, including 2 cases of C3–C4; 7 cases of C4–C5; 16 cases of C5–C6; 13 cases of C6–C7; 36 cases in the control group, including 3 cases of C3–C4; 8 cases of C4–C5; 14 cases of C5–C6; and 11 cases of C6–C7; there was no significant difference between the two groups in demographics, duration of symptoms, operation stage (*p* > 0.05), and operation time (80.5 ± 5.5 vs. 81.5 ± 3.5 min). The VAS in the upper limbs pain was significantly higher postoperatively than preoperatively in both groups (*p* < 0.05). The cocktail group had a lower VAS than the control group 1 week postoperatively (Fig. 3) (*p* < 0.05); however, VAS not different between groups at the remaining time points (Fig. 4). The NDI scores were significantly better postoperatively than preoperatively (Fig. 5), and no significant differences were seen when comparing nodes at postoperative follow-up (*p* > 0.05). All patients successfully completed the operation, and no total spinal cord anesthesia, dural tear, motor dysfunction, or infection complications were found. In the control group, two cases with foraminal stenosis were found to have unrelieved pain in the early postoperative period, but the pain was relieved at the final follow-up and did not convert to open decompression surgery. At the final follow-up, the modified MacNab criteria showed that there were 27 cases of excellent; 9 cases of good; 2 cases of fair; and 0 cases of poor status in the cocktail group and 25 cases of excellent; 8 cases of good; 3 cases of fair; and 0 cases of poor status in the control group.

## Discussion

For cervical disc herniation, especially for patients with foraminal stenosis, postoperative nerve root dysfunction is more common and mainly manifests as pain, numbness, or weakness in upper limbs. The causes of its occurrence are multifactorial. Choi et al. [9] described a case of motor paralysis after C4-5 posterior cervical foraminotomy, arguing that C5 nerve roots usually cover the entire intervertebral disc space and, therefore, require more excessive retraction than other nerve roots. Youn et al. [10] suggested that removing a herniated disc or bone spur could lead to a potential risk of motor paralysis due to excessive retraction, while retraction without discectomy could increase the risk of transient root injury. Lee et al. [11] reported transient motor weakness and transient sensory changes after PCF and believed that excessive pulling, mechanical damage, and thermal damage during drilling were the reasons for weakness or sensory changes after posterior cervical foraminotomy/PECD. We believed that the continuous mechanical pressure from the protruding nucleus pulposus tissue preoperatively and the release of tumor necrosis factor alpha caused by it activate neutrophils and monocytes to produce a local inflammatory response [12]. Additionally, the mechanical pulling and stimulation of nerve roots and damage of surrounding soft tissues during the operation lead to the generation and release of inflammatory substances and increase the permeability of surrounding capillaries, further aggravating local inflammation and edema. These inflammatory factors also stimulate the exudation of nerve roots and ganglia, possibly leading to nerve root edema and ischemia, and ultimately causing ischemia–reperfusion injury [12–14]. Makkar and Zhang [15, 16] reported that epidural steroid injections provided significant pain relief and reduced disability in lumbar spine. Shin et al. [17] reported that the application of epidural steroids in PELD significantly reduced postoperative back pain and lower limb pain and promoted the early functional recovery of patients because steroids can alleviate inflammatory response by inhibiting the chemotactic aggregation of inflammatory cells, adhesion of leukocytes, and release of histamine and kinase [18]. DU et al. [12] introduced the use of gelatin sponge infiltration steroids and ropivacaine to cover nerve roots during lumbar MIS-TLIF surgery and achieved good early analgesic effects. Research by Yang et al. [7] confirmed that in PELD, the use of cocktail therapy (dexamethasone, ropivacaine, and vitamin B12) and the placement of nerve roots after infiltration with gelatin sponge can more effectively relieve early postoperative nerve root dysfunction and promote postoperative recovery.

To prevent nerve root edema and irritation after PPECD and avoid the risk of total spinal anesthesia (TSA) and spinal cord injury, epidural administration is not available [19, 20]. The use of DSM for the local infiltration of nerve roots is an emerging method to achieve the aforementioned safety improvements. It is advantageous in that it can use a cocktail treatment of multiple drugs and maintain a high drug concentration and sustained release in the early stage. Additionally, unlike PELD, where gelatin sponge is placed between disc and dorsal dura, we placed gelatin sponge on the dorsal side of the nerve root to prevent epidural hematoma. Due to the limited intraoperative resection scope of the ligamentum flavum, it can play a barrier role, reduce the possibility of drugs entering the dural sac, and improve safety. This study uses gelatin sponge as a raw material biofilm to act as DSM. It has the characteristics of degradability, anti-adhesion, and reduction in disordered hyperplasia of peripheral scars.

Ropivacaine is a long-acting amide local anesthetic, which has low cardiotoxicity and neurotoxicity [21]. Low concentrations of ropivacaine (10 mg/h) has obvious sensory block but no motor block [22]. Dexmedetomidine can effectively reduce the ischemia–reperfusion injury of local tissues, better protect the damaged nerve tissue, and speed up the repair. Topical dexmedetomidine exhibits anti-inflammatory effects against local acute inflammatory reactions by reducing the production of inflammatory cytokines. Moreover, dexmedetomidine as an adjuvant can prolong the action time of ropivacaine and enhance the analgesic effect [23–25]. Vitamin B12 can reduce abnormal nerve discharge and promote nerve regeneration [26]. Dexamethasone is known to reduce the production of prostaglandins and participate in analgesia with anti-inflammatory effects. It can inhibit the secretion of neuropeptides from small nerve fibers, thereby reducing pain. Simultaneously, it can reduce edema, fibrin formation, telangiectasia, white blood cell aggregation, as well as the proliferation of capillaries and fibroblasts, scarring, and so on [27]. Therefore, these four drugs loaded at the gelatin sponge have a close synergistic effect on anti-inflammatory, analgesic, and nerve growth.

In this study, the early pain of the cocktail group was significantly relieved within 1 week postoperatively compared with the control group. However, the single-center, retrospective design of this study and small sample size are its main limitations. Further prospectively designed randomized controlled trials to verify our results are warranted. The proportion of mixed drugs was based on clinical experience. These aspects should be further explored to ensure optimal clinical outcomes. To further explore the sensitive factors affecting postoperative analgesia and compare the differences between single drug usage, the further multiple regression analysis was needed.

## Conclusion

Cocktail treatment, in which a drug sustained-release material made of gelatin sponge was impregnated with dexamethasone, ropivacaine, dexmedetomidine and vitamin B12, facilitates pain relief and early postoperative recovery after PPECD.

## Data Availability

The datasets generated during the current study are public at the email liao_b@hotmail.

## Abbreviations

CDH: Cervical disc herniation
ACDF: Anterior cervical decompression and fusion
PPECD: Posterior percutaneous endoscopic cervical discectomy
PELD: Percutaneous endoscopic lumbar disectomy
VASs: Visual analog scores
NDI: Neck disability index

## References

[1] Carette S, Fehlings MG (2005). Clinical practice. Cervical radiculopathy. N Engl J Med.

[2] Haijun M, Xiaobing Z, Bin G (2020). Trans-interlamina percutaneous endoscopic cervical discectomy for symptomatic cervical spondylotic radiculopathy using the new Delta system. Sci Rep.

[3] Xiao CM, Yu KX, Deng R (2019). Modified K-hole percutaneous endoscopic surgery for cervical foraminal stenosis: partial pediculectomy approach. Pain Physician.

[4] Quillo-Olvera J, Lin GX, Kim JS (2018). Percutaneous endoscopic cervical discectomy: a technical review. Ann Transl Med.

[5] Wan Q, Zhang D, Li S (2018). Posterior percutaneous full-endoscopic cervical discectomy under local anesthesia for cervical radiculopathy due to soft-disc herniation: a preliminary clinical study. J Neurosurg Spine.

[6] Yang JS, Chu L, Chen L, Chen F, Ke ZY, Deng ZL (2014). Anterior or posterior approach of full-endoscopic cervical discectomy for cervical intervertebral disc herniation? A comparative cohort study. Spine (Phila Pa 1976).

[7] Yang JS, Liu KX, Chu L (2020). Cocktail treatment with a gelatin sponge impregnated with ropivacaine, dexamethasone, and vitamin B12 promotes early postoperative recovery after percutaneous endoscopic lumbar discectomy: a retrospective case-controlled study. Pain Physician.

[8] Ahn Y (2019). Current techniques of endoscopic decompression in spine surgery. Ann Transl Med.

[9] Choi KC, Ahn Y, Kang BU, Ahn ST, Lee SH (2013). Motor palsy after posterior cervical foraminotomy: anatomical consideration. World Neurosurg.

[10] Youn MS, Shon MH, Seong YJ, Shin JK, Goh TS, Lee JS (2017). Clinical and radiological outcomes of two-level endoscopic posterior cervical foraminotomy. Eur Spine J.

[11] Lee U, Kim CH, Chung CK (2018). The recovery of motor strength after posterior percutaneous endoscopic cervical foraminotomy and discectomy. World Neurosurg.

[12] Du JP, Fan Y, Hao DJ, Huang YF, Zhang JN, Yuan LH (2018). Application of gelatin sponge impregnated with a mixture of 3 drugs to intraoperative nerve root block to promote early postoperative recovery of lumbar disc herniation. World Neurosurg.

[13] Hamm-Faber TE, Aukes H, van Gorp EJ, Gültuna I (2015). Subcutaneous stimulation as an additional therapy to spinal cord stimulation for the treatment of low back pain and leg pain in failed back surgery syndrome: four-year follow-up. Neuromodulation.

[14] Wang W, Atianjoh F, Gauda EB, Yaster M, Li Y, Tao YX (2011). Increased expression of sodium channel subunit Nav1.1 in the injured dorsal root ganglion after peripheral nerve injury. Anat Rec (Hoboken).

[15] Makkar JK, Gourav KKP, Jain K (2019). Transforaminal versus lateral parasagittal versus midline interlaminar lumbar epidural steroid injection for management of unilateral radicular lumbar pain: a randomized double-blind trial. Pain Physician.

[16] Zhang Y, Yang XJ, Zeng TH, Qiu YY, Wang YT, Liang FG (2017). A retrospective study of epidural and intravenous steroids after percutaneous endoscopic lumbar discectomy for large lumbar disc herniation. Chin J Traumatol.

[17] Shin SH, Hwang BW, Keum HJ, Lee SJ, Park SJ, Lee SH (2015). Epidural steroids after a percutaneous endoscopic lumbar discectomy. Spine (Phila Pa 1976).

[18] Coutinho AE, Chapman KE (2011). The anti-inflammatory and immunosuppressive effects of glucocorticoids, recent developments and mechanistic insights. Mol Cell Endocrinol.

[19] Bernards CM, Kopacz DJ, Michel MZ (1994). Effect of needle puncture on morphine and lidocaine flux through the spinal meninges of the monkey in vitro. Implications for combined spinal-epidural anesthesia. Anesthesiology.

[20] Wu W, Yan Z (2018). Intraoperative total spinal anesthesia as a complication of posterior percutaneous endoscopic cervical discectomy. Eur Spine J.

[21] Djerada Z, Feliu C, Cazaubon Y (2018). Population pharmacokinetic-pharmacodynamic modeling of ropivacaine in spinal anesthesia. Clin Pharmacokinet.

[22] Axelsson K, Johanzon E, Essving P, Weckström J, Ekbäck G (2005). Postoperative extradural analgesia with morphine and ropivacaine. A double-blind comparison between placebo and ropivacaine 10 mg/h or 16 mg/h. Acta Anaesthesiol Scand.

[23] Talke P, Stapelfeldt C, Lobo E, Brown R, Scheinin M, Snapir A (2005). Effect of alpha2B-adrenoceptor polymorphism on peripheral vasoconstriction in healthy volunteers. Anesthesiology.

[24] Sukegawa S, Higuchi H, Inoue M, Nagatsuka H, Maeda S, Miyawaki T (2014). Locally injected dexmedetomidine inhibits carrageenin-induced inflammatory responses in the injected region. Anesth Analg.

[25] Kaye AD, Chernobylsky DJ, Thakur P (2020). Dexmedetomidine in enhanced recovery after surgery (ERAS) protocols for postoperative pain. Curr Pain Headache Rep.

[26] Altun I, Kurutaş EB (2016). Vitamin B complex and vitamin B12 levels after peripheral nerve injury. Neural Regen Res.

[27] Gurbet A, Bekar A, Bilgin H, Korfali G, Yilmazlar S, Tercan M (2008). Pre-emptive infiltration of levobupivacaine is superior to at-closure administration in lumbar laminectomy patients. Eur Spine J.

